# Denervation drives skeletal muscle atrophy and induces mitochondrial dysfunction, mitophagy and apoptosis via miR-142a-5p/MFN1 axis

**DOI:** 10.7150/thno.40857

**Published:** 2020-01-01

**Authors:** Xiaofan Yang, Pingping Xue, Hongrui Chen, Meng Yuan, Yu Kang, Dominik Duscher, Hans-Günther Machens, Zhenbing Chen

**Affiliations:** 1 Department of Hand Surgery, Union Hospital, Tongji Medical College, Huazhong University of Science and Technology, Wuhan 430022, China.; 2 Department of Pharmacy, Tongji Hospital, Tongji Medical College, Huazhong University of Science and Technology, Wuhan 430030, China.; 3 Department of Plastic and Hand Surgery, Technical University of Munich, Munich 81675, Germany.

**Keywords:** denervation, skeletal muscle atrophy, miRNA-142a-5p, MFN1, mitophagy, apoptosis

## Abstract

**Rationale**: Peripheral nerve injury is common in clinic, which leads to severe atrophy and dysfunction of the denervated muscles, but the underlying mechanism is not fully understood. Recent studies advanced the causative role of mitochondrial dysfunction in muscle atrophy, while the upstream triggers remained unclear.

**Methods**: In the present study, Atrophy of gastrocnemius and tibialis anterior (TA) were evaluated in mice sciatic nerve transection model. Transmission electron microscopy (TEM) was then used to observe the microstructure of atrophic gastrocnemius and mitochondria. Subsequently, small RNA sequencing, luciferase reporter assay and Electrophoretic Mobility Shift (EMSA) were performed to explore the potential signaling pathway involved in skeletal muscle atrophy. The effects of the corresponding pathway on mitochondrial function, mitophagy, apoptosis and muscle atrophy were further determined in C2C12 cells and denervated gastrocnemius.

**Results**: Gastrocnemius and TA atrophied rapidly after denervation. Obvious decrease of mitochondria number and activation of mitophagy was further observed in atrophic gastrocnemius. Further, miR-142a-5p/ mitofusin-1 (MFN1) axis was confirmed to be activated in denervated gastrocnemius, which disrupted the tubular mitochondrial network, and induced mitochondrial dysfunction, mitophagy and apoptosis. Furthermore, the atrophy of gastrocnemius induced by denervation was relieved through targeting miR-142a-5p/MFN1 axis.

**Conclusions**: Collectively, our data revealed that miR-142a-5p was able to function as an important regulator of denervation-induced skeletal muscle atrophy by inducing mitochondrial dysfunction, mitophagy, and apoptosis via targeting MFN1. Our findings provide new insights into the mechanism of skeletal muscle atrophy following denervation and propose a viable target for therapeutic intervention in individuals suffering from muscle atrophy after peripheral nerve injury.

## Introduction

Denervation of skeletal muscle results in a rapid and programmed loss in muscle size and performance, termed muscle atrophy. Previous researches considered it a result of protein homeostasis lacking. However, the molecular mechanisms that govern the imbalance between pathways controlling protein synthesis and degradation in denervated muscle atrophy remained to be explored, notwithstanding the large amount of work done 1,2. In addition, some studies attributed this process to apoptosis of muscle cells, yet the mechanisms governing such apoptosis remain uncertain 3,4. Recently, mitochondrial dysfunction has been shown to play a pivotal role in the process of muscle atrophy, with evidence of alterations in mitochondrial biogenesis, mitochondrial respiration, and mitochondrial dynamics following prolonged skeletal muscle unloading, while the triggers of these changes in mitochondria remain to be explored 5,6.

Mitochondria are organelles that produce the vast majority of cellular energy through the process of oxidative phosphorylation (OXPHOS). Beyond this metabolic role, however, mitochondria also play central roles in diverse processes such as programmed cell death, autophagy, redox signaling, and Ca^2+^ homeostasis 7. Mitochondrial dynamics are characterized by frequent fusion and division of mitochondria within cells. The balance between fusion and division is required for mitochondria to regulate various physiological processes 8. For example, mitochondrial dynamics change during the cellular response to stress. In some conditions that adversely impact cellular health, such as nutrient limitation or modest inhibition of cytosolic protein synthesis, the mitochondrial network becomes highly interconnected, which facilitates ATP production and promotes cell survival 9. Mitochondrial dynamics are also integrated into cell cycle progression and cell death pathways, placing them at the heart of cellular life and death decisions 10, 11. As such, loss or dysfunction of the mitochondrial fusion or division machines are broadly confirmed in neurodegenerative diseases, heart failure, diabetes and cancer 12-15. In some cases, disease associated changes in mitochondrial dynamics can be attributed to altered expression of the mitochondrial fusion and division related proteins (e.g. MFN1/2 and Drp1). In other cases, aberrant signaling pathways are predicted to alter mitochondrial dynamics. In either case, aberrant mitochondrial dynamics is associated with mitochondrial dysfunction, contributing to disease pathology. Skeletal muscle is rich of mitochondria, which are necessary for its contractile activity and metabolism. Recent researches have shown the alterations of mitochondrial dynamics in atrophic skeletal muscle, but the causal relationship between these alterations and muscle atrophy remains unclear 16, 17.

MicroRNAs (miRNAs) are a class of noncoding RNAs that are approximately 22 nucleotides (nt) in length and are important regulators of gene expression. miRNAs are involved in diverse physiological and pathological processes, including cell proliferation, differentiation, apoptosis, autophagy, tumorigenesis, and even epigenetic regulation 18-21. The expression of miRNAs is regulated by many factors associated with various environmental stresses, such as starvation, hypoxia, inflammation, oxidative stimulation and denervation 22-24. In addition, numbers of studies have recently reported the regulatory effect of miRNAs on mitochondrial fission, fusion and mitophagic protein expression in skeletal muscle and other tissues 25,26, but whether miRNAs specifically regulate denervated skeletal muscle atrophy through mitochondrial mechanisms remains to be studied.

In the present study, gastrocnemius and TA showed a rapid loss in muscle mass in the unilateral sciatic nerve transection model over the first two weeks. Significant decrease of mitochondria number and activation of mitophagy were observed by TEM. Then small RNA sequencing was carried out and miRNA-142a-5p was confirmed to be up-regulated in atrophic gastrocnemius. We hypothesized that miRNA-142a-5p was a critical regulator of mitochondrial dynamics by targeting MFN1 and the resulting disruption of mitochondria dynamics promoted atrophy of denervated skeletal muscle. To test this hypothesis, miRNA-142a-5p mimic was transfected into C2C12 cells, MFN1 was then down-regulated, extensive mitochondrial fragmentation, depolarization of mitochondrial membrane potential (Δψm) and accumulation of reactive oxygen species (ROS) were observed, activation of mitophagy and apoptosis of skeletal muscle cells were also confirmed in C2C12 cells. Importantly, these effects were attenuated by overexpression of MFN1. In mice sciatic nerve transection models, up-regulation of miRNA-142a-5p achieved similar results and aggravated muscle atrophy, while down-regulation of miRNA-142a-5p or overexpression of MFN1 were both able to relieve atrophy. Overall, this study indicates that miR-142a-5p is an important regulator of denervation-induced skeletal muscle atrophy by inducing mitochondrial dysfunction, mitophagy, and apoptosis via targeting MFN1.

## Materials and methods

### Animal procedures

Ten-week old male C57BL/6J mice were housed on a 24 hours day-night cycle and purchased from the Animal Experiment Center of Huazhong University of Science and Technology. Denervation was performed surgically on the right hind legs of mice as previously described 27. The mice were randomized into the following groups (5 mice/group): sham operation group (control); denervation group; denervation + miR-142a-5p agomir (Ribobio, China) group; denervation + miR-142a-5p antagomir (Ribobio) group; denervation + rAAV-MFN1 (Hanbio Biotechnology, China) group; denervation + miR-142a-5p agomir + rAAV-MFN1 group. 5 nmol agomir or 10 nmol antagomir in 0.1 ml saline buffer was locally injected into gastrocnemius once every 3 days for 2 weeks. rAAV-MFN1 injection of gastrocnemius was performed 3 weeks in advance to build MFN1 overexpression model. Specifically, 10 µl of virus (1.2 × 10^12^ vg/ml) were injected into each point around gastrocnemius. The syringe was not removed until 5-10 min after the end of infusion to allow diffusion of the virus. 4-6 injections were performed for each mouse (Figure S1A, Figure S2A). Mice were euthanized at the indicated time, gastrocnemius and tibialis anterior (TA) were removed, weighed and frozen for the next experiments.

All experimental procedures were in accordance with the guidelines of the Chinese National Institutes of Health. Experimental protocols were approved by the Ethical Committee on Animal Experiments (Huazhong University of Science and Technology).

### Cell culture

Briefly, C2C12 cells (mouse myoblast cell line; iCell Bioscience Inc., China) were grown in high-glucose Dulbecco's modified Eagle's medium (DMEM; Gibco, USA) supplemented with 10% fetal bovine serum (FBS; Gibco, USA), 100 U/ml of penicillin, and 100μg/ml of streptomycin in a 5% CO_2_ humidified atmosphere at 37 °C.

### Wet weight measurement, hematoxylin-eosin (HE) staining and fiber diameter quantification

Both the operational and contralateral sides of the gastrocnemius and TA were collected and weighed. The wet weight ratio was defined as the muscle weight of the operational side divided by that of the contralateral side.

For HE staining, the samples were fixed with paraformaldehyde (4%), dehydrated, and paraffin embedded. Cross-sectional 4-μm thick slices of the muscle were prepared and stained with HE (Bioyear, China) to observe the pathological changes of atrophy.

To measure fiber diameters, gastrocnemius sections with immunofluorescence staining of wheat germ agglutinin (WGA) were analyzed with fibers being selected manually. The ImageJ software was used to quantify the minimal Feret's diameter for each.

### Western blot

Frozen gastrocnemius and TA tissues were homogenized in RIPA buffer containing 1mM PMSF, and Protease Inhibitor Cocktail (Roche, New Jersey). Lysates were centrifuged for 20 min at 12,000 × g (4°C). Supernatants were transferred to a separate tube, and the BCA assay (Beyotime, China) was used for protein level quantification. Proteins were separated by SDS-PAGE gels (Beyotime), transferred to PVDF membranes (Millipore, USA), and blots were blocked for 1 h with 5% nonfat dry milk in TBS at room temperature. Primary antibodies against the following targets were proceeded overnight at 4°C: mouse anti- myosin heavy chain (MHC; 1:3,000; R&D Systems, USA), rabbit anti-TOM20 (1:10000; Abcam, UK), rabbit anti-TIM23 (1:1000; Abcam), rabbit anti-MFN1 (1:1000; Abcam), rabbit anti-PTEN induced putative kinase 1 (PINK1; 1:1000; Abcam), mouse anti-Parkin (1:2000; Abcam), rabbit anti-LC3B (1:500; Abcam), rabbit anti-Bax (1:5000; Abcam), rabbit anti-Bcl-2 (1:2000; Abcam), rabbit anti-cleaved-caspase 9 (1:1000; Cell Signaling Technology, USA), rabbit anti-cleaved-caspase 3 (1:2000; Cell Signaling Technology), rabbit anti-cytochrome c (1:5000; Abcam), mouse anti-GAPDH (1:5000; Proteintech, China) and rabbit anti-COX IV (1:1000; Proteintech). After 3 washes, blots were incubated with appropriate secondary antibodies (Abcam) at room temperature for 1 hour. An ECL detection reagent and X-ray film were used for protein detection. Similar procedures were carried out for C2C12 myotubes.

### Transmission electron microscopy (TEM)

Mitochondrial morphology in gastrocnemius was analyzed by TEM. Gastrocnemius were trimmed into approximately 1.0 mm^3^ strips and fixed in 2.5% glutaraldehyde for 18-20 h, followed by 1% osmium tetroxide for 1 h. Then, the tissues were dehydrated, and embedded in epoxy. Ultra-thin sections (60 nm thick) were cut, and the sections were stained with 2% uranyl acetate and lead citrate and observed using a transmission electron microscope (Hitachi, Japan). A minimum of 10 photomicrographs were taken randomly from each sample. The number of mitochondria was counted on micrographs in a blinded fashion as previously described 28.

### Small RNA sequencing

Two weeks post-operation, three mice were sacrificed and samples (normal gastrocnemius from contralateral side and atrophic gastrocnemius from operational side) were immediately frozen using liquid nitrogen. Total RNA was isolated from samples using the Trizol (Invitrogen) according to the manufacturer's protocol. Libraries of small RNAs were constructed using TruSeq Small RNA Library Preparation Kits (Illumina, USA). The libraries were sequenced by Illumina HiSeq 2000 at RiboBio. Differential expression was assessed by DEseq using read counts as input. The Benjamini-Hochberg multiple test correction method was enabled. Differentially expressed miRNAs were chosen according to the criteria of adjusted p-value < 0.05.

### Cell transfection

The miRNA-142a-5p mimic, miRNA-142a-5p inhibitor, and the relevant negative controls (mimic control and inhibitor control) were commercially obtained from Ribobio company. The plasmids for overexpressing MFN1 (pcDNA3.1-EF1α-MFN1-3flag-CMV-GFP) and the negative control plasmid (pcDNA3.1-EF1α-mcs-3flag-CMV-GFP) were purchased from Hanbio Biotechnology (Figure S1B, Figure S2B). Cell transfection was performed with the Lipofectamine 2000 reagent (Invitrogen) according to the manufacturer's protocol. At 48 h after transfection, the cells were processed for in vitro assays.

### Dual luciferase reporter gene assay

The possible miR-142a-5p binding sites in the MFN1 gene 3'‑untranslated region (UTR) were predicted by bioinformatics analysis using TargetScan version 7.2 online tool (http://www.targetscan.org/vert_72/). HEK-293T cells were seeded and cultured on 24-well plates. Then, cells were co-transfected with the miRNAs (mimic control, miR-142a-5p mimic, inhibitor control or miR-142a-5p inhibitor) and pGL3-luciferase promoter vector containing the WT or MUT 3'UTR of MFN1. After 48 hours, cells were collected and luciferase was detected using Dual Luciferase Reporter Assay (Promega Corporation, USA). The firefly luciferase signal was normalized to that of the renilla luciferase signal.

### Electrophoretic Mobility Shift Assay (EMSA)

The validation of miRNA-mRNA interactions was performed using the Molecular Probes' fluorescence-based EMSA Kit (Invitrogen) according to the manufacturer's protocol. For the binding assays, the following oligonucleotides were designed and used: miR-142a-5p; MFN1-UTR, a 23-mer RNA sequence for the 3'UTR corresponding to MFN1 with the target site for miR-142a-5p; anti-miR-142a-5p, a modified antisense oligodeoxynucleotide complementary to the sequence of miR-142a-5p; anti-miR-142a-5p MIS, an antisense oligodeoxynucleotide containing 11 mismatches compared to anti-miR-142a-5p. All oligonucleotides were purchased from Sigma-Aldrich, and their specific sequences were listed in Figure 3F. The corresponding oligonucleotides were incubated in binding buffer (750mM KCl, 0.5 mM dithiothreitol, 0.5 mM EDTA, 50 mM Tris, pH 7.4) for 30 min at 37 ℃, and the reaction products were then separated on a 10% non-denaturing polyacrylamide gel. After staining the gel with SYBRE® Green solution for 20 min in the dark, photos were taken with 300 nm UV transillumination.

### Visualization of mitochondrial and lysosome in C2C12 cells

Cells were grown on coverslips inside a cell culture dish. After treatment, the culture medium in the dish was replaced with prewarmed staining solution containing 200 nM MitoTracker™ Green FM probe (Thermo Fisher Scientific, USA). Cells were incubated with the staining solution for 30 min at 37 °C. To visualize lysosome, cells were then incubated in a prewarmed staining solution containing 25 nM LysoTracker™ Red DND-99 probe for the next 10 min. After staining, the cells were washed two times with PBS and incubated with fresh media. Then, the cells were visualized using a Zeiss confocal laser scanning microscope (Carl Zeiss, LSM 800). The results were obtained from at least 30 cells in each group.

### Measurement of mitochondrial membrane potential

Δψm was assessed in C2C12 cells using the JC-1 probe (Beyotime). Cells were incubated with JC-1 staining solution for 20 min at 37 °C. Images were obtained using a confocal laser scanning microscopy. The red and green fluorescence intensities were quantified using the Zeiss LSM Image Examiner software. Red fluorescence represented JC-1 aggregates, whereas green fluorescence represented the monomeric form of JC-1. Δψm was reflected by the ratio of red to green fluorescence intensity. The results were obtained from at least 30 cells in each group.

### Measurement of ROS and mitochondrial-derived O_2_^∙-^ levels in C2C12 cells

Total levels of cellular ROS in C2C12 cells were detected using the fluorescent probe DCFH-DA (Beyotime) according to the manufacturer's instructions. For the analysis of mitochondrial O_2_^•-^, C2C12 cells were labeled with MitoSOX Red, a highly selective fluorescent probe, for the detection of O_2_^•-^ generated within mitochondria. Briefly, cells were cultured in a cell culture dish and were treated as indicated. MitoSOX Red reagent stock solution was diluted in HBSS/Ca/Mg buffer to generate a 5 μM reagent working solution. Cells were then incubated with 5 μM MitoSOX Red reagent working solution in the dark at 37 °C for 10 min. After three washes with warm buffer, live imaging was performed using a confocal laser scanning microscope. The results were obtained from at least 30 cells in each group.

### Measurement of mitochondrial respiratory chain complexes activities

Mitochondria were isolated from gastrocnemius or C2C12 cells using Mitochondria Isolation Kits (Solarbio, China) according to the manufacturer's protocol. The activities of complexes I, II, IV and ATP synthase (V) were measured with the MitoProfile Rapid Microplate Assay Kit (Abcam) according to the manufacturer's instructions. Complexes I, II, IV, and V were immune-captured in the wells of microplates, and enzymatic activity was measured with a kinetic colorimetric assay kit (Thermo Fisher Scientific). The total and mitochondrial protein concentrations were determined using the BCA assay (Beyotime).

### Cell death ELISA

Gastrocnemius were extracted, blotted to remove excess liquid, minced, and homogenized. Differential centrifugation was then used to isolate cytosolic extracts and protease inhibitors were added to prevent protein degradation. A cell death detection ELISA (Roche, USA) was used to quantitatively determine the apoptotic DNA fragmentation by measuring the cytosolic histone-associated mono- and oligonucleosomes. The assay was performed according to the manufacturer's instructions. Final absorbance was determined with a microplate reader at 405 nm. Data were normalized to total protein concentration by BCA assay. A wavelength of 490 nm was used as the reference wavelength.

### Statistical analysis

The GraphPad Prism 5 software (GraphPad Software, USA) was used for all analyses. Results are shown as mean ± standard deviation (SD) for at least three independent experiments. ANOVA with a post-hoc Dunnett's test was used for all comparisons. P < 0.05 was the threshold of statistical significance.

### Supplementary Methods

Immunofluorescence, Quantitative real-time PCR (qRT-PCR), Autophagy detection with Ad-GFP-LC3B, TUNEL staining, flow cytometry and Total Antioxidant Capacity Analyses are available in supplementary materials.

## Results

### Denervation led to atrophy and mitochondrial disorder of denervated muscles

**Figure 1:**
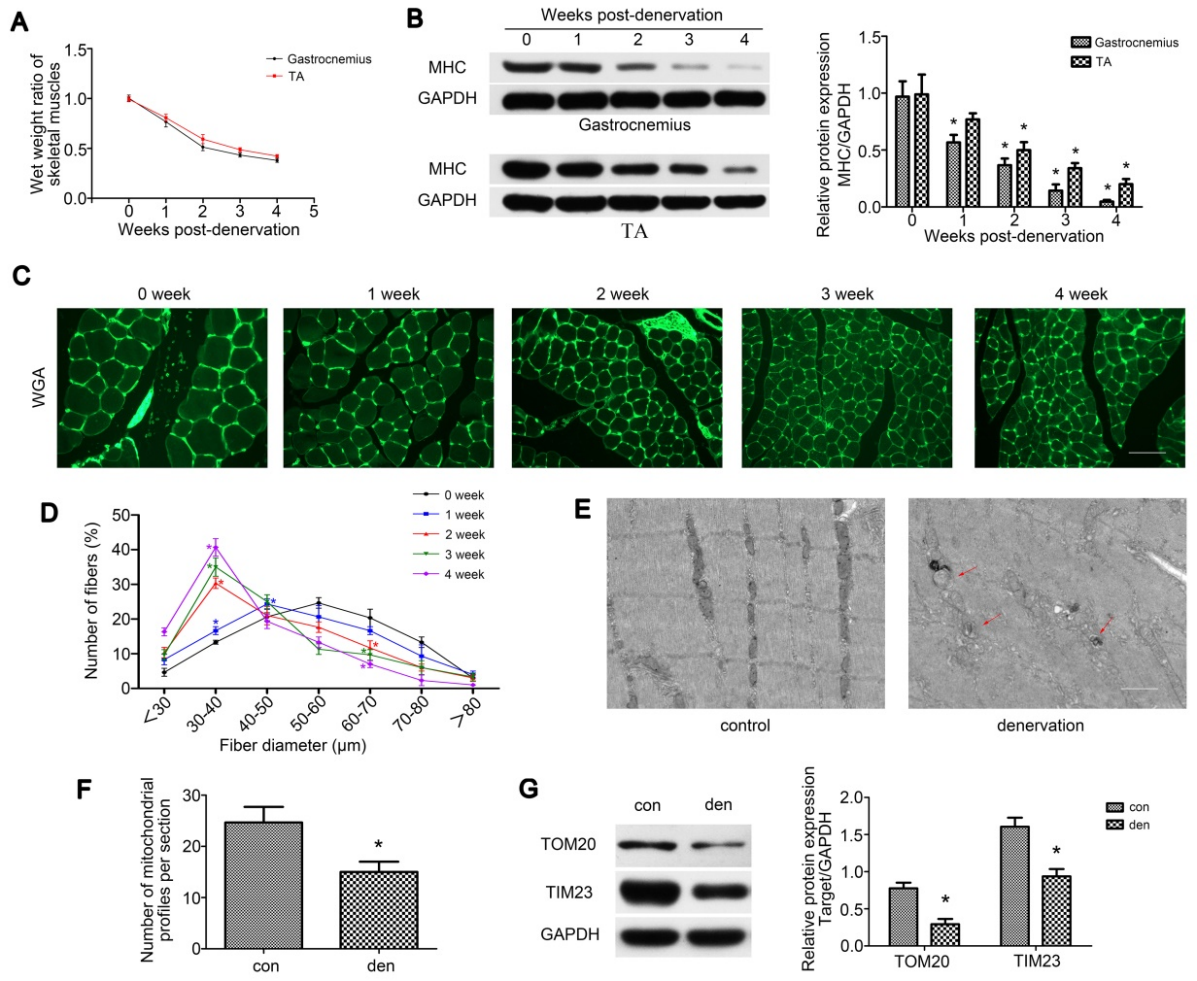
** Denervation led to atrophy and mitochondrial disorder of denervated muscles.** (A) The wet weight ratio (the weight of the operational side divided by the contralateral side) of gastrocnemius and TA at indicated time points post-denervation. (B) Western blot analysis of the dynamic changes of MHC protein expression after denervation. Relative grey values analyses were performed. (C, D) Quantification of gastrocnemius fibers diameter by immunofluorescence staining of WGA. Scale bar 50 µm. (E, F) Microstructure of gastrocnemius and mitochondria was observed by TEM. Red arrows indicated autophagosomes enclosing mitochondria. The number of mitochondrial profiles were statistically analysed. Scale bar 1.25 µm (G) Western blot analysis of the mitochondrial outer/inner membrane protein TOM20/TIM23. Data were presented as mean ± SD. n=5. *P < 0.05 vs control (0 week). Den, denervation; Con, control.

To explore the process of muscle atrophy after denervation, gastrocnemius and TA were analyzed in mice sciatic nerve transection model. Consistent with previous research 29, TA and gastrocnemius of the operation side rapidly atrophied after denervation surgery. Weight measurements revealed that the atrophic process was biphasic, with a rapid loss (average 49% in gastrocnemius) in muscle mass over the first 2 weeks and then a more gradual reduction (average 13% in gastrocnemius) over the following 2 weeks (Figure 1A). As skeletal muscle atrophy was usually accompanied by decreased expressions of sarcomeric proteins, we tracked the levels of MHC after denervation by western blot, which showed a consistent trend with muscle weight (Figure 1B). We further analyzed fiber diameter through WGA staining and observed the gradual diminution in muscle fiber size over time after denervation, showing a shift towards fiber of a lesser diameter (Figure 1C-D). As the atrophy of denervated muscles occurred primarily during the first two weeks post-denervation, this time point was targeted in the following experiments. To observe the morphology changes of denervated gastrocnemius, TEM was carried out and the atrophic gastrocnemius was found to be disorganized, showing some broken Z-lines, degraded M-lines and irregular intermyofibrillar space (Figure 1E). Besides, more autophagosomes enclosing mitochondria (mitophagosomes, marked by red arrows) and obviously fewer mitochondria could be found in atrophic gastrocnemius than the contralateral side (Figure 1E-F). Western blot analysis of the mitochondrial outer/inner membrane protein TOM20/TIM23 also indicated that mitochondria were reduced in atrophic gastrocnemius (Figure 1G). Totally, these results showed that the denervated skeletal muscle atrophied over time in a biphasic manner, during which mitophagy was activated and mitochondria decreased.

### Small RNA sequencing and target predicting

**Figure 2:**
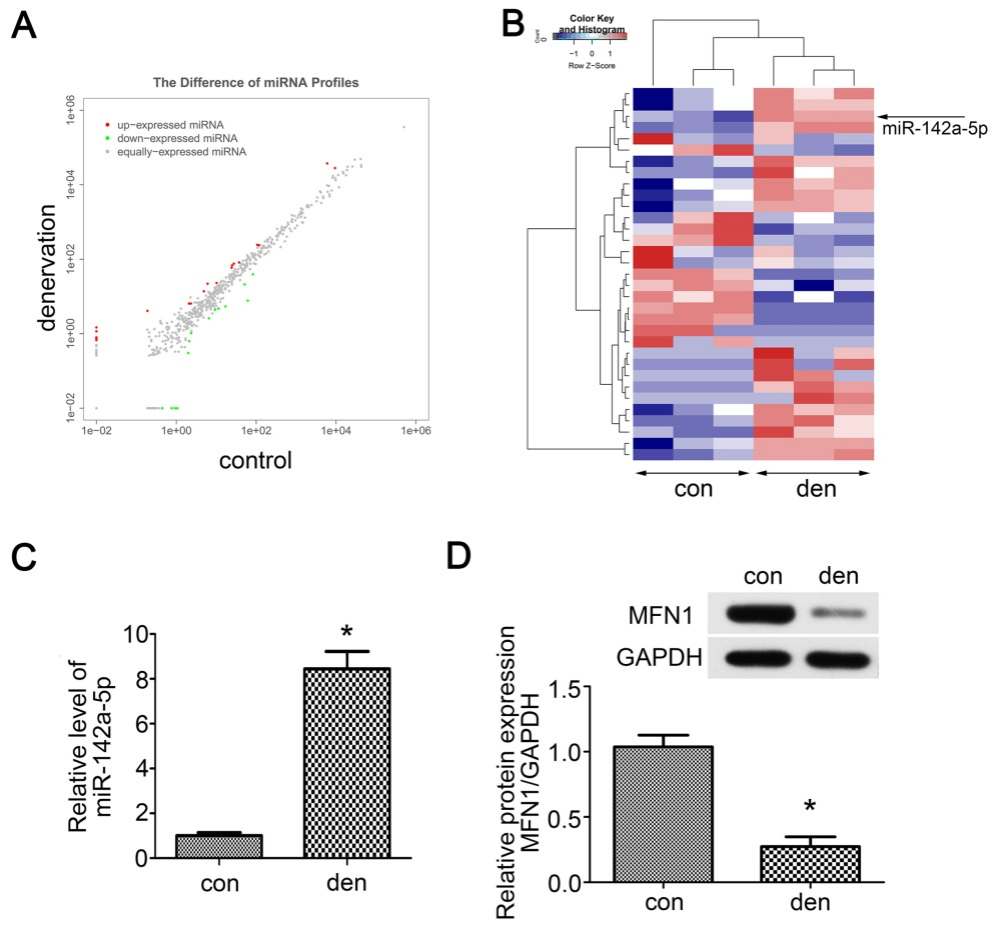
** Small RNA sequencing and target predicting.** (A) Scatter plot of miRNAs expression in denervated and contralateral gastrocnemius two weeks post-operation. The red and green point in the plot represented the differentially expressed miRNAs showing statistical significance (P < 0.05). (B) Hierarchical cluster analysis of the significantly upregulated and downregulated miRNAs (P < 0.05). Each column represented a sample and each row represented a miRNA. The expression levels were presented in different colors indicating expression levels above and below the median expression level across all samples. miR-142a-5p was marked by arrow. (C) qRT-PCR was performed to verify the small RNA sequencing results of miR-142a-5p in gastrocnemius of contralateral side and denervation side. (D) MFN1, the predicted target of miR-142a-5p, was detected by western blot. Data were presented as mean ± SD. n=3. *P < 0.05 vs contralateral side. Den, denervation; Con, control.

**Figure 3:**
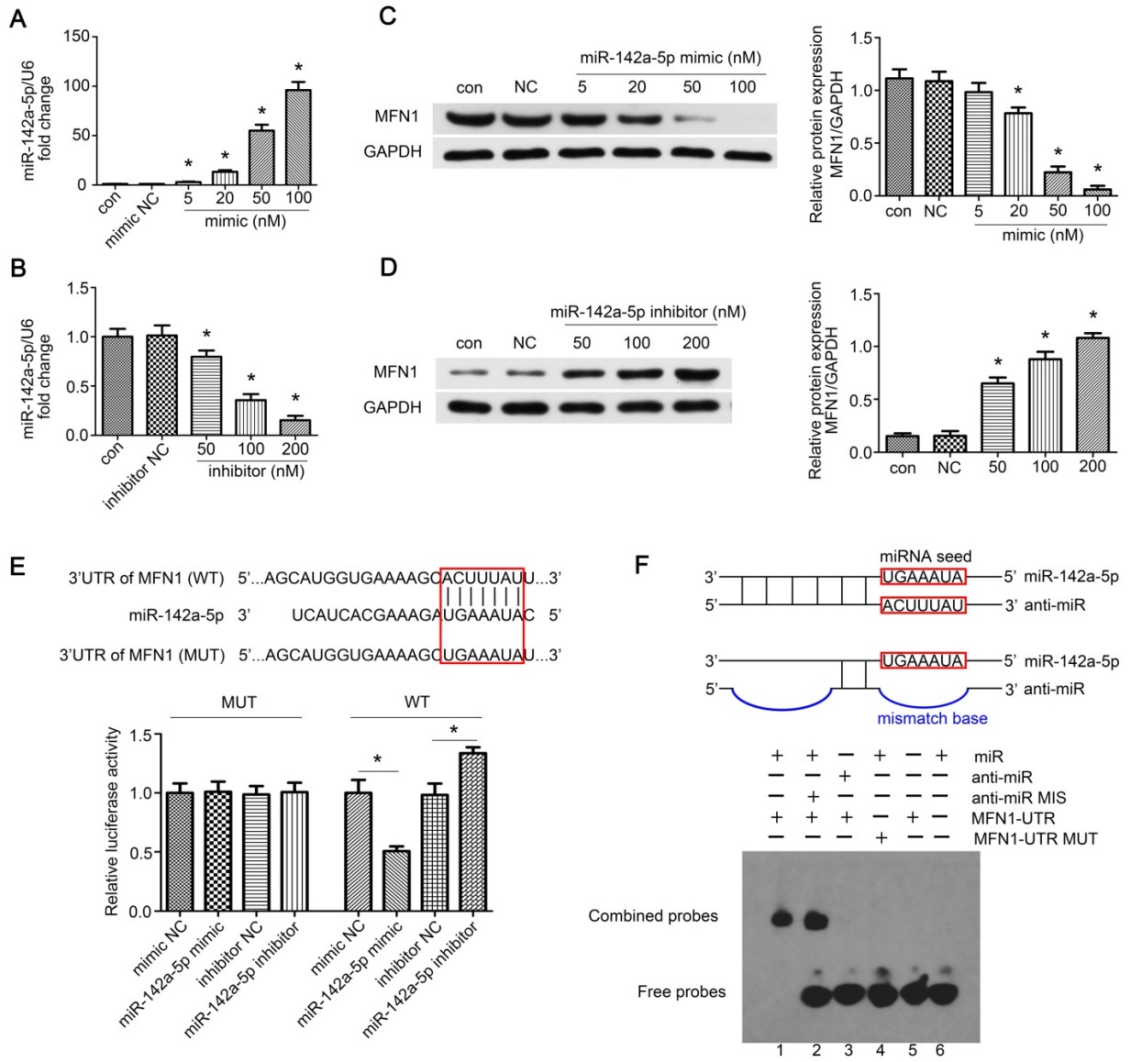
** MFN1 was a direct target of miR-142a-5p.** (A, B) Concentration gradients of miR-142a-5p mimic and inhibitor were introduced to permit efficient up- or down- regulation of miR-142a-5p levels in C2C12 cells. (C, D) Western blotting analysis of MFN1 expression in C2C12 cells transfected with concentration gradients of miR-142a-5p mimics/inhibitors. (E) Schematic drawing of the putative binding sites or mutations of miR-142a-5p in MFN1 mRNA 3'UTR. 293T cells were co-transfected with miRNA and luciferase reporter containing wild type (WT) 3'UTR of MFN1 (pGL3-3'UTR of MFN1-WT) or mutant (MUT) 3'UTR of MFN1 (pGL3-3'UTR of MFN1-MUT), and luciferase activity was detected at 48 h post-transfection. (F) EMSA was performed to confirm the interaction of MFN1 mRNA and miR-142a-5p. RNA and DNA oligonucleotides were designed according to the model and corresponded to the incubation of the probes for EMSA on a non-denaturing gel. Lane 1 represented the binding and competition between miR-142a-5p and the MFN1 3'UTR. Data were presented as mean ± SD. *P < 0.05 vs NC. NC, negative control; Con, control.

As miRNAs are vital to mitochondrial homeostasis, small RNA sequencing was performed to identify potential miRNAs or signaling pathways involved in skeletal muscle atrophy. In total, 33 differentially expressed miRNAs were identified (Table S1), in which 19 miRNAs were upregulated and 14 miRNAs were downregulated (Figure 2A). The Hierarchical cluster analysis showed the differentially expressed miRNAs (adjusted p-value < 0.05) between atrophied gastrocnemius and control (Figure 2B). To further confirm the above results, qRT-PCR was performed and miR-142a-5p, which was previously reported to play roles in myocardial infarction and Alzheimer's disease, was found to be up-regulated by nearly 8-fold in denervated gastrocnemius (Figure 2C). To determine whether the increase of miR-142a-5p after denervation was fiber type-specific, FISH was performed in extensor digitorum longus (EDL, a typically fast-twitch muscle mainly containing type IIB and IID fibers), soleus (SOL, a typically slow-twitch muscle mainly containing type I and IIA fibers) and gastrocnemius (mixed type muscle), all of which achieved a higher fluorescence intensity compared to control (Figure S3). Potential miR-142a-5p targets were then predicted using bioinformatics analysis in order to seek out the downstream mechanisms governing gastrocnemius atrophy, the results revealed that MFN1 was a conserved target of miR-142a-5p with the pairing position of 1940-1946 in MFN1 3'UTR (Figure 3E, Data from TargetScan). Given that MFN1 is important for both mitochondrial dynamics and cell apoptosis, the expression level of MFN1 was further assessed via western blot, revealing significant downregulation of MFN1 in atrophic gastrocnemius relative to the contralateral side (Figure 2D). Together these results revealed the differentially expressed miRNAs in atrophic gastrocnemius, in which miR-142a-5p might play key role in muscle atrophy by targeting MFN1.

### MFN1 was a direct target of miR-142a-5p

To further confirm the above hypothesis, concentration gradients of miR-142a-5p mimic and inhibitor were transfected into C2C12 cells. We found that 100 nM mimic greatly enhanced exogenous miR-142a-5p expression whereas 200 nM inhibitor led to a significant decrease compared to the negative control (Figure 3A-B). Moreover, MFN1 levels in miR-142a-5p mimic transfected C2C12 cells decreased in a dose-dependent fashion, while inhibitor constructs resulted in increased MFN1 protein levels (Figure 3C-D). Then the luciferase reporters (pGL3-3'UTR of MFN1-WT and MFN1-MUT) were constructed and transfected into the 293T cells, it was seen that overexpression of miR-142a-5p suppressed the luciferase activity of pGL3-MFN1-WT while miR-142a-5p knockdown achieved the opposite effect. Overexpression or knockdown of miR-142a-5p both exerted no significant effects on the luciferase activity of pGL3-MFN1-MUT in 293T cells (Figure 3E). EMSA was also performed to further validate the miRNA-mRNA interactions, the specific binding of miR-142a-5p and MFN1-UTR oligonucleotides was clearly seen in Lane 1, while the bindings were not evident in anti-miR-142a-5p/MFN1-UTR (Lane 3) and miR-142a-5p/MFN1-UTR MUT (Lane 4) oligonucleotides (Figure 3F). Briefly, these data indicated that miR-142a-5p could inhibit MFN1 expression by binding to the MFN1 mRNA 3'UTR.

### miR-142a-5p/MFN1 axis regulated mitochondrial morphology and function in vitro

**Figure 4:**
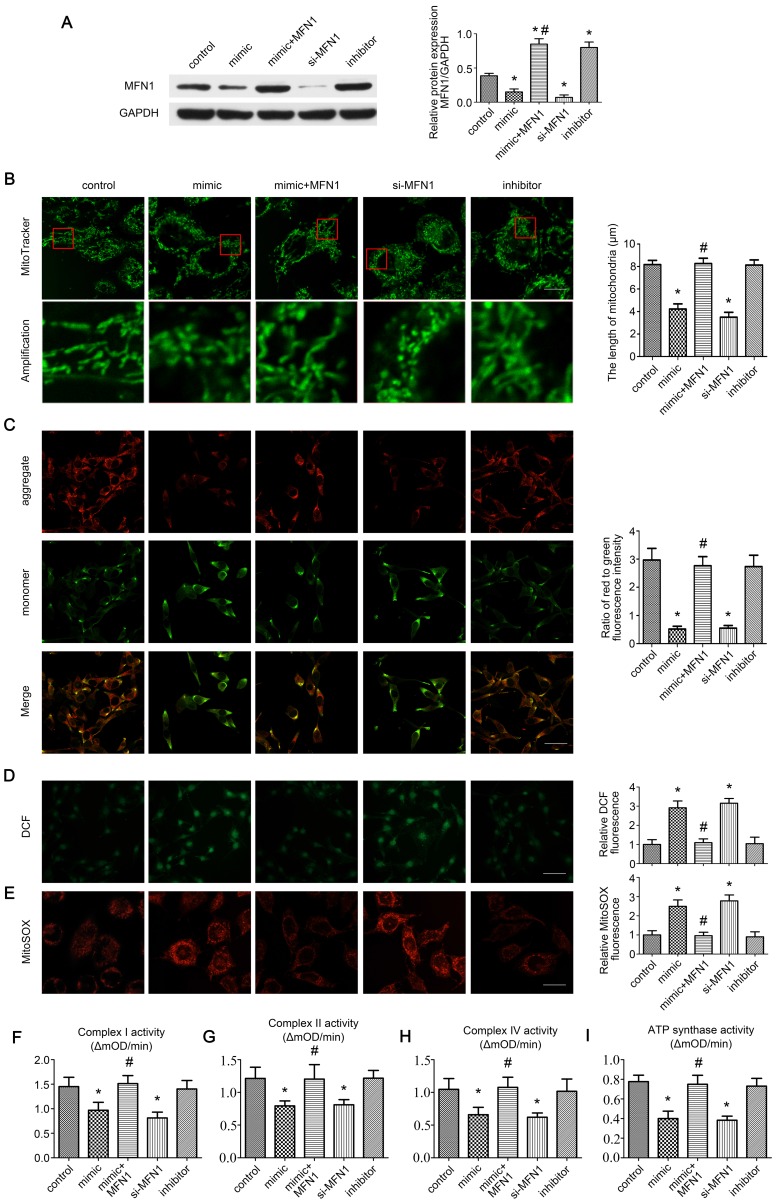
** miR-142a-5p/MFN1 axis regulated mitochondrial morphology and function in vitro.** (A) Western blotting analysis confirmed the effect of C2C12 transfection on MFN1. (B) The morphology of the mitochondria was labeled by MitoTracke Green probes. Part of the photographs (red box) were amplified. The length of mitochondria in each group was analysed. Scale bar 10 µm. (C) Determination of Δψm using the JC-1 probe. Red and green fluorescence represented the aggregate and monomeric form of JC-1 respectively. The merged images indicated co-localization of JC-1 aggregates and monomers. The ratio of red to green fluorescence intensity was calculated. Scale bar 50 µm. (D) Intracellular ROS levels were detected using the fluorescent probe DCFH-DA. Relative DCF fluorescence was analysed. Scale bar 50 µm. (E) The mitochondrial O_2_^•-^ levels were detected in C2C12 cells labeled with MitoSOX Red. Relative MitoSOX fluorescence was analysed. Scale bar 25 µm. (F, G, H, I) Activities of complexes I, II, and IV and ATP synthase. Data were presented as mean ± SD. *P < 0.05 vs control. ^#^P < 0.05 vs mimic. mOD, mitochondrial optical density.

As MFN1 is vital for mitochondrial dynamics 8, we further explored the effect of miR-142a-5p/MFN1 axis on mitochondria in C2C12 cells. Firstly, the expressions of MFN1 were detected in cells under different treatment, revealing that miR-142a-5p mimic and si-MFN1 transfection significantly repressed MFN1 expression, whereas the miR-142a-5p inhibitor promoted MFN1 expression. In addition, transfection of MFN1 overexpressing plasmid rescued the reduced MFN1 content induced by miR-142a-5p mimic and achieved a higher level of MFN1 than control (Figure 4A). Then the mitochondrial morphology in each group was observed by MitoTracker Green probes, it was obvious that mitochondria displayed tubular network with elongated and filamentous morphology in the control group (8.17 ± 0.38 μm), whereas miR-142a-5p mimic (4.23 ± 0.45 μm) and si-MFN1 (3.50 ± 0.44 μm) transfection markedly altered this morphology, resulting in highly fragmented mitochondria and a discontinuous network. Overexpression of MFN1 disrupted mimic-induced excessive mitochondrial fragmentation and increased the filamentous mitochondria (8.27 ± 0.47 μm). Cells in miR-142a-5p inhibitor group exhibited similar mitochondrial morphology (8.13 ± 0.45 μm) to control. A quantitative analysis of mitochondrial morphology was obtained by measuring the length of the mitochondria in each group (Figure 4B).

Δψm is necessary for mitochondrial oxidative phosphorylation and ATP production 30. The effect of miR-142a-5p/MFN1 axis on Δψm in C2C12 cells was determined by the JC-1 probe. As shown in Figure 4C, miR-142a-5p mimic and si-MFN1 transfection decreased the ratio of red to green fluorescence, indicating the depolarization of Δψm, while MFN1 overexpression abolished the mimic-induced dissipation of Δψm. Transfection of miR-142a-5p inhibitor showed no obvious influence on Δψm.

Mitochondria are primary sources of ROS within cells. Mitochondrial dysfunction with reduced activities of the mitochondrial respiratory chain complexes result in the overproduction of mitochondrial ROS (mtROS), which can induce oxidative damage to DNA, proteins and lipids not only in mitochondria but also in other cellular compartments. This can in turn exacerbate mitochondrial dysfunction and cause further damage to cells and tissues 31, 32. Here the intracellular ROS levels were accessed using the fluorescent probe DCFH-DA. It was obvious that miR-142a-5p mimic and si-MFN1 transfection markedly increased cellular ROS levels, whereas MFN1 overexpression alleviated the accumulation of ROS caused by miR-142a-5p mimic and reached a similar ROS level to control (Figure 4D). As the superoxide anion (O_2_^•-^) generated in mitochondria is the primary ROS, we next assessed mitochondrial O_2_^•-^ levels in C2C12 cells using MitoSOX Red in order to investigate the effect of the miR-142a-5p/MFN1 axis on mtROS production.

Results were similar to the DCF fluorescence findings (Figure 4E), further demonstrating that miR-142a-5p/MFN1 axis could regulate cellular ROS level by targeting mitochondria. Considering that overproduction of mtROS often originated from the reduced activities of mitochondrial respiratory chain complexes, we further investigated these complexes (I, II, IV and ATP synthase) and got a similar trend to the above results (Figure 4F-I).

Together, these results confirmed that the miR-142a-5p/MFN1 axis was able to regulate mitochondrial morphology and function in vitro.

### miR-142a-5p/MFN1 axis activated mitophagy in vitro

**Figure 5:**
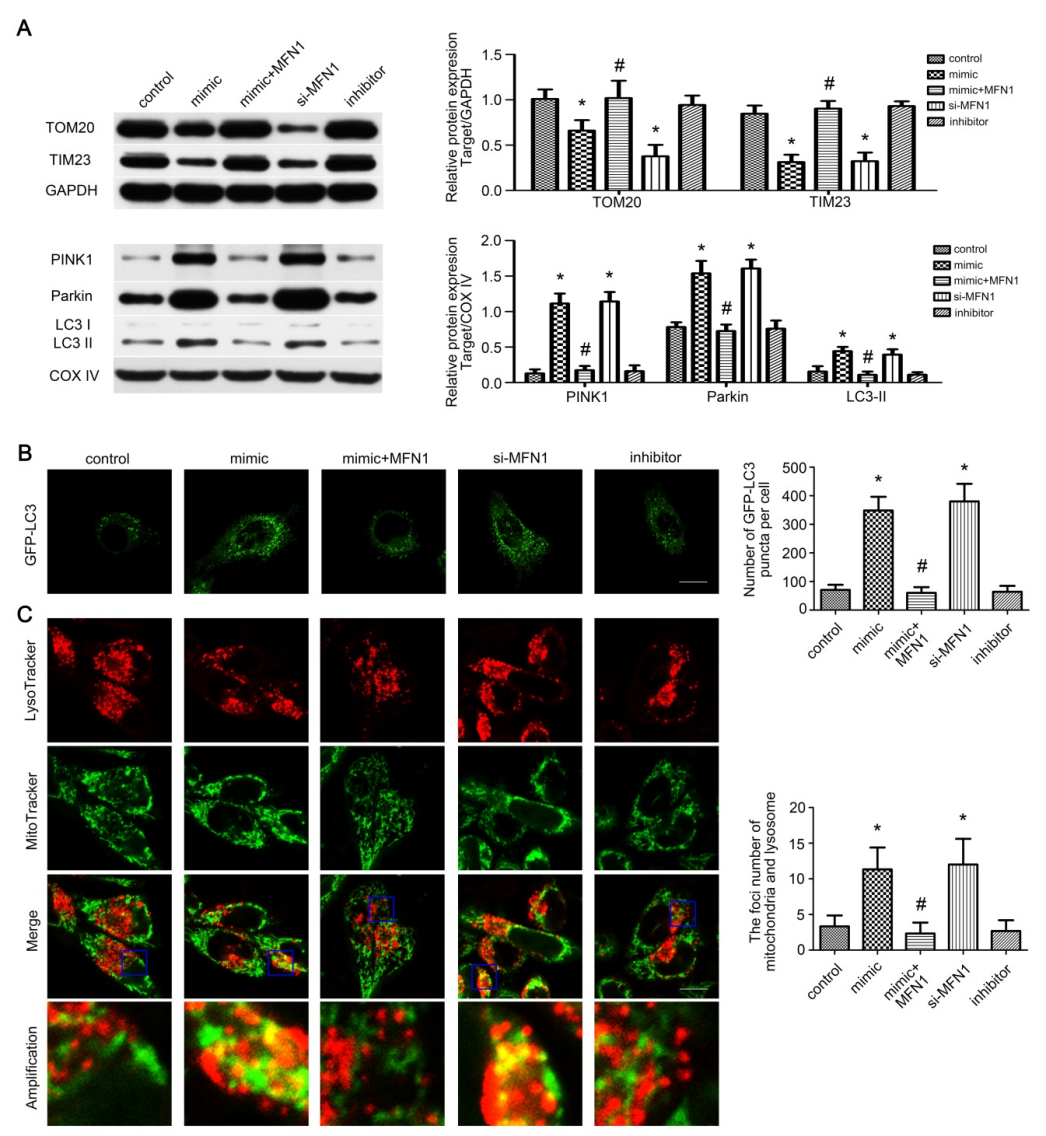
** miR-142a-5p/MFN1 axis activated mitophagy in vitro.** (A) Western blotting analysis of the mitophagy parameters in C2C12 cells. The expressions of total TOM20, TIM23 and mito-PINK1, Parkin, LC3II were evaluated separately. GAPDH and COX IV were used as internal references. (B) Representative images of C2C12 cells expressing GFP-LC3 in each group. Quantification of the average number of GFP-LC3 puncta per cell. Scale bar 10 µm. (C) Co-staining of mitochondria and lysosome by MitoTracke Green and LysoTracker Red. Part of the photographs (blue box) were amplified. The number of the overlaps between the mitochondria and lysosome was counted to quantify the mitophagy activity. Scale bar 10 µm. Data were presented as mean ± SD. *P < 0.05 vs control. ^#^P < 0.05 vs mimic.

As mitophagy is involved in mitochondrial quantity and quality control 33, we wonder whether it is activated by miR-142a-5p/MFN1 axis. Western blot revealed that miR-142a-5p mimic and si-MFN1 transfection repressed total TOM20, TIM23 expressions and increased mito-LC3II, PINK1 and Parkin expressions, confirming the activation of mitophagy in C2C12 cells through PINK1/Parkin pathway. In addition, MFN1 overexpression could block the protein changes induced by miR-142a-5p mimic (Figure 5A). Subsequently, GFP-LC3 was transfected into C2C12 cells and the average number of GFP-LC3 puncta per cell reflected the magnitude of autophagic activity. Similarly, miR-142a-5p mimic and si-MFN1 transfection significantly increased fluorescence puncta, which was then rescued by up-regulation of MFN1 (Figure 5B). To provide more solid evidence for the action of miR-142a-5p/MFN1 axis on mitophagy, mitochondria and lysosome were co-stained after transfection. As shown in Figure 5C, spindle mitochondria were separated from the lysosomes in the control group. Following miR-142a-5p mimic and si-MFN1 transfection, however, mitochondria divided into several fragmentations, which were widely tagged by lysosomes, indicative of mitophagy activation. In brief, the above results indicated that mitophagy was regulated by miR-142a-5p/MFN1 axis in vitro.

### miR-142a-5p/MFN1 axis dominated C2C12 cells apoptosis

**Figure 6:**
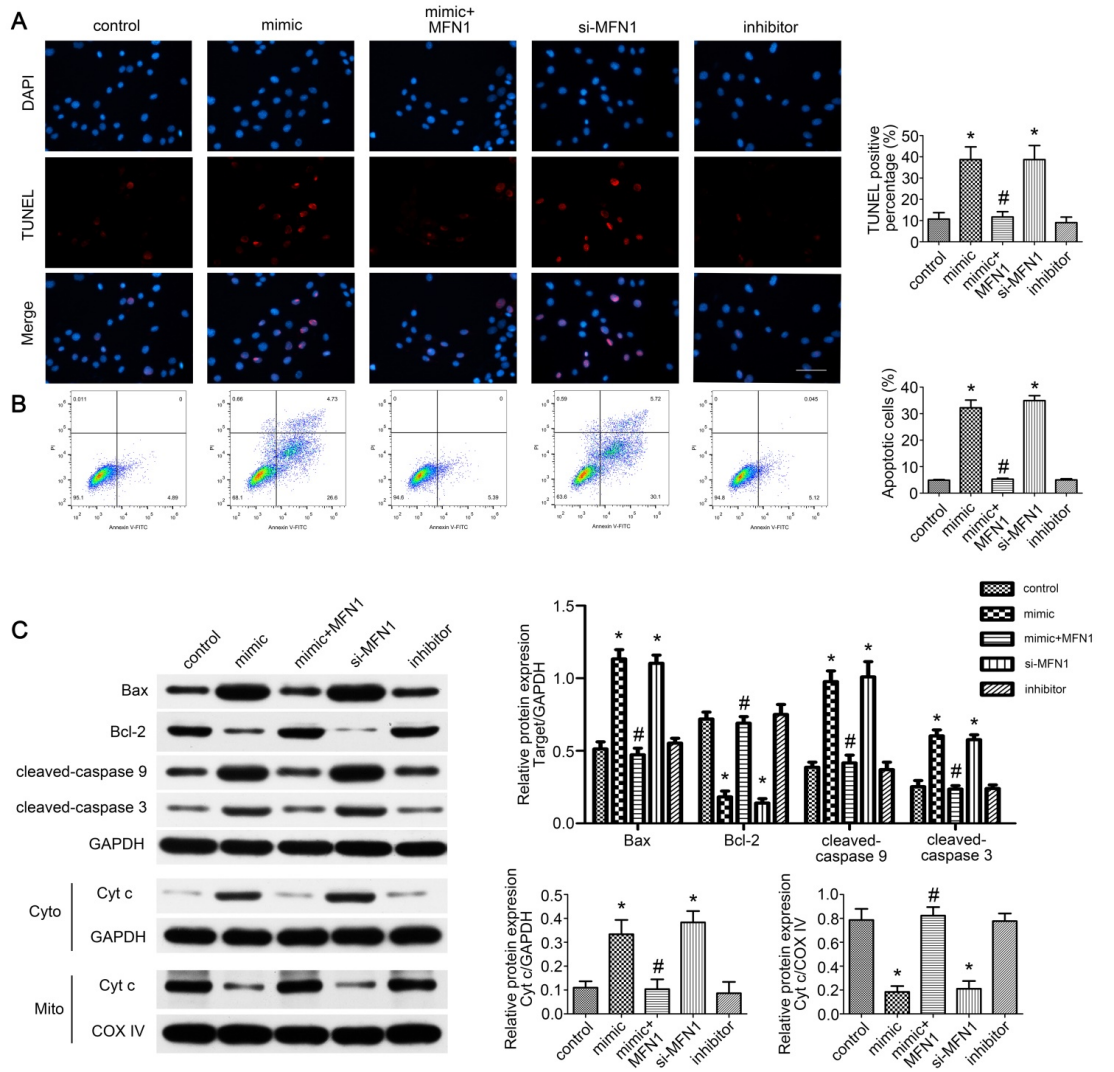
** miR-142a-5p/MFN1 axis dominated C2C12 cells apoptosis.** (A) TUNEL assay indicated the apoptosis of C2C12 cells in each group. Positive percentage was analysed. Scale bar 50 µm. (B) FCM analysis of the apoptosis of C2C12 cells. The apoptotic rate was further analysed. (C) Western blot analysis of the protein changes related to mitochondria-mediated apoptosis. Cytochrome c in cytosolic and mitochondrial subfractions was assessed respectively. Data were presented as mean ± SD. *P < 0.05 vs control. ^#^P < 0.05 vs mimic. Cyto, cytoplasm; Mito, mitochondria; Cyt c, cytochrome c.

Given the role of mitofusin proteins in mitochondria-mediated apoptosis 34,35, we further evaluated the effect of miR-142a-5p/MFN1 axis on apoptosis of C2C12 cells. From TUNEL assay and FCM results, we found that miR-142a-5p mimic and si-MFN1 transfection significantly promoted apoptosis of C2C12 cells while MFN1 overexpression eliminated this effect of miR-142a-5p mimic and achieved a low level of apoptosis (Figure 6A-B). Western blotting was also carried out to analyze the expression of apoptosis signaling pathway-associated proteins. As shown in Figure 6C, miR-142a-5p mimic and si-MFN1 transfection was associated with significantly higher levels of pro-apoptotic cleaved caspase-3, cleaved caspase-9, and Bax, as well as with lower levels of anti-apoptotic Bcl-2 relative to control. The situation in MFN1overexprssion group was similar to control, thus indicating the abolition of the proapoptotic effect of miR-142a-5p mimic. As mitochondria-mediated apoptosis is characterized by cytochrome c liberation from mitochondria into cytoplasm, the cytosolic and mitochondrial levels of cytochrome c were then assessed respectively. In line with other findings, miR-142a-5p mimic and si-MFN1 transfection groups exhibited higher levels of cytosolic cytochrome c and lower levels of mitochondrial cytochrome c, while up-regulation of MFN1 reversed the effect of miR-142a-5p mimic. miR-142a-5p inhibitor group showed similar cytochrome c expression patterns to control. The above results well demonstrated the apoptosis regulating effect of miR-142a-5p/MFN1 axis in C2C12 cells.

### Restoring MFN1 expression relieved miR-142a-5p mediated atrophy of denervated gastrocnemius

**Figure 7:**
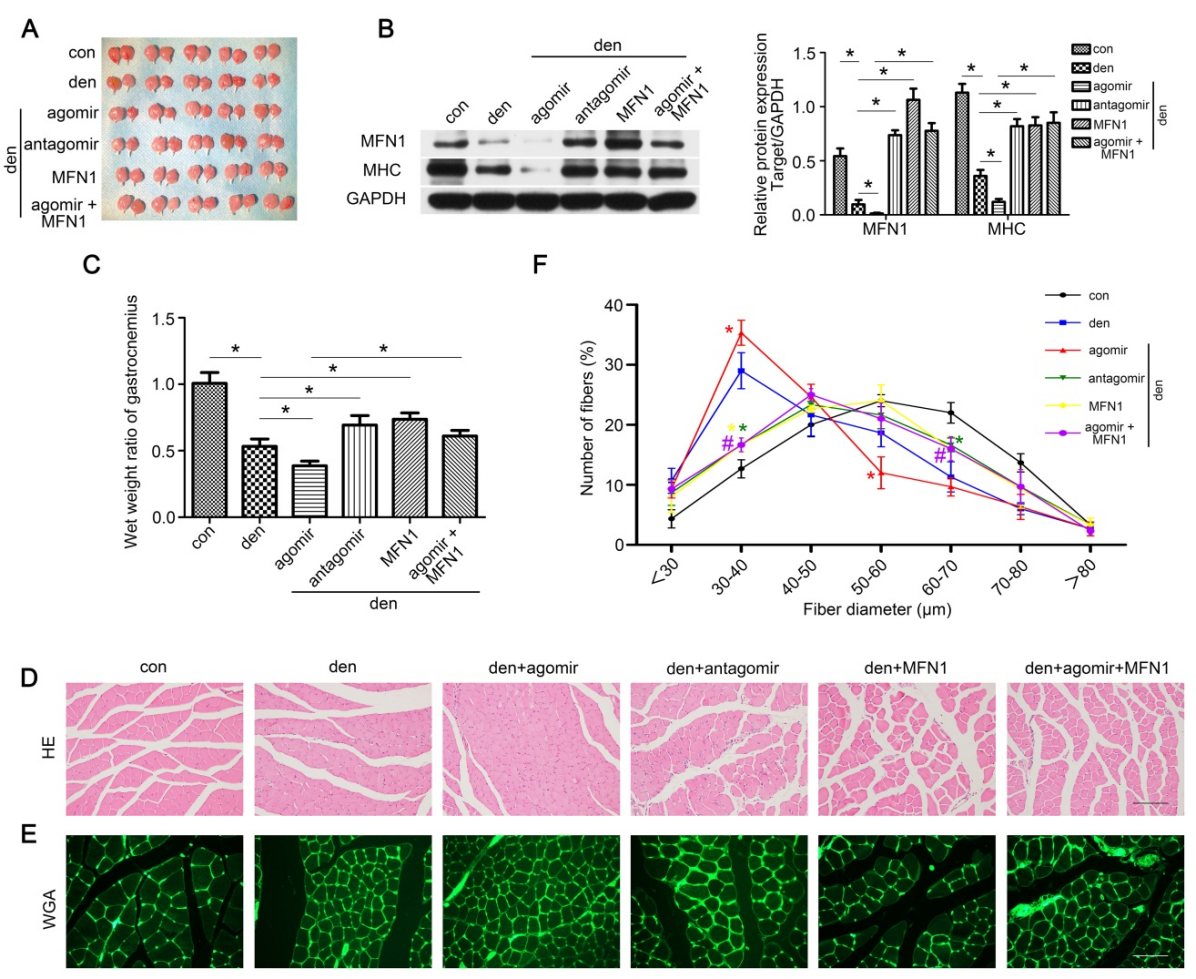
** Restoring MFN1 expression relieved miR-142a-5p mediated atrophy of denervated gastrocnemius.** (A) Gastrocnemius of different groups harvested 2 weeks post-denervation. (B) Western blotting analysis confirmed the expressions of MFN1 and MHC. (C) The wet weight ratio of gastrocnemius in different groups. (D) Morphological observation of gastrocnemius muscles in different groups by HE staining. Scale bar 50 μm. (E, F) Quantification of muscle fibers diameter by immunofluorescence staining of WGA. Scale bar 50 µm. Data were presented as mean ± SD. n=5. *P < 0.05 (vs den). ^#^P < 0.05 vs den + agomir group. Den, denervation; Con, control.

To clarify the role of the miR-142a-5p/MFN1 axis in the atrophy of denervated gastrocnemius, miR-142a-5p/MFN1 inhibition and overexpression models were generated as detailed in the Methods section, and gastrocnemius samples were harvested 2 weeks post-denervation (Figure 7A). The expression of MFN1 was confirmed by western blot (Figure 7B). Muscle mass evaluation, MHC expression and HE staining revealed that miR-142a-5p agomir significantly aggravated muscle atrophy, whereas this was reversed by the injection of rAAV-MFN1. Furthermore, miR-142a-5p antagomir administration alleviated the atrophy of denervated gastrocnemius (Figure 7B-D). We further analyzed fiber diameter and observed that diminution in muscle fiber size was induced by miR-142a-5p agomir and could be reversed by rAAV-MFN1injection. miR-142a-5p antagomir administration attenuated the atrophy of denervated gastrocnemius and produced a shift towards a larger fiber diameter than that of the denervation group (Figure 7E-F). These results indicated that miR-142a-5p/MFN1 axis mediated the atrophy of denervated gastrocnemius.

### MFN1 overexpression preserved mitochondrial function in denervated gastrocnemius

**Figure 8:**
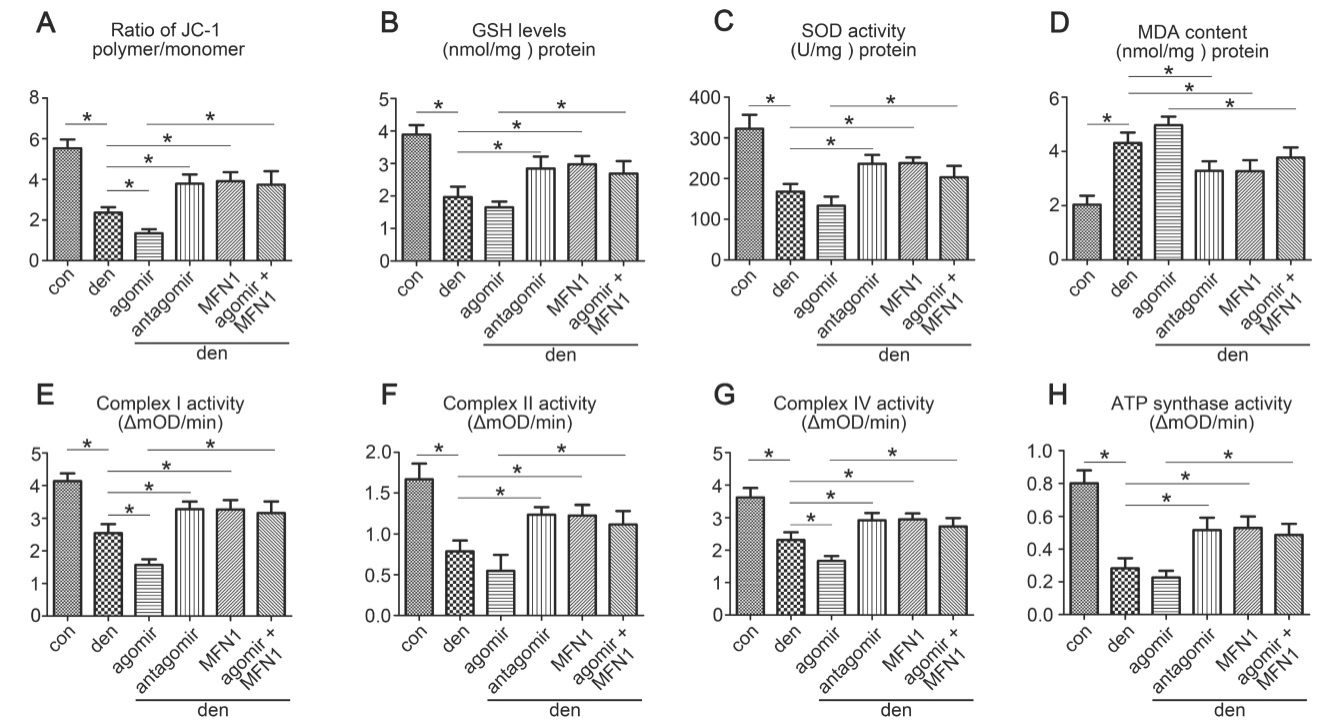
** MFN1 overexpression preserved mitochondrial function in denervated gastrocnemius.** (A) Determination of Δψm using the JC-1 probe. The ratio of polymer/monomer fluorescence intensity was calculated. (B, C, D) The content of anti-oxidant factors, GSH and SOD, and the lipid peroxide MDA in gastrocnemius of different group. (E, F, G, H) Activities of complexes I, II, and IV and ATP synthase. Data were presented as mean ± SD. n=5. *P < 0.05. Den, denervation; Con, control. mOD, mitochondrial optical density.

To investigate the effect of miR-142a-5p/MFN1 axis on mitochondrial function, Δψm, antioxidant capacity and mitochondrial respiratory chain complexes activities were evaluated. The results showed that miR-142a-5p agomir aggravated the depolarization of Δψm in denervated gastrocnemius, whereas miR-142a-5p antagomir and rAAV-MFN1 had the opposite effect. In addition, rAAV-MFN1 injection was able to reverse the effect of miR-142a-5p agomir on gastrocnemius Δψm (Figure 8A). In addition to the changes in Δψm, levels of anti-oxidants such as GSH and SOD, as well as the lipid peroxide MDA were also detected to evaluate oxidative stress in gastrocnemius. As shown in Figure 8B-D, denervation and miR-142a-5p agomir treatment induced a decline in GSH and SOD levels and an increase in MDA levels, indicating increased oxidative stress. Administration of miR-142a-5p antagomir and rAAV-MFN1 both relieved denervation-induced oxidative stress (as characterized by increased GSH and SOD levels and decreased MDA levels). In addition, we further found that rAAV-MFN1 administration could alleviate the oxidative stress caused by denervation + miR-142a-5p agomir treatment. Similarly, the activities of mitochondrial respiratory chain complexes were significantly reduced in denervated gastrocnemius and improved upon the administration of miR-142a-5p antagomir and rAAV-MFN1. rAAV-MFN1 was able to further protect the activities of complexes from denervation + miR-142a-5p agomir administration (Figure 8E-H). The above results therefore indicated that MFN1 overexpression reversed mitochondrial dysfunction induced by miR-142a-5p in denervated gastrocnemius.

### Restoring MFN1 expression attenuated miR-142a-5p induced mitophagy and apoptosis in denervated gastrocnemius

**Figure 9:**
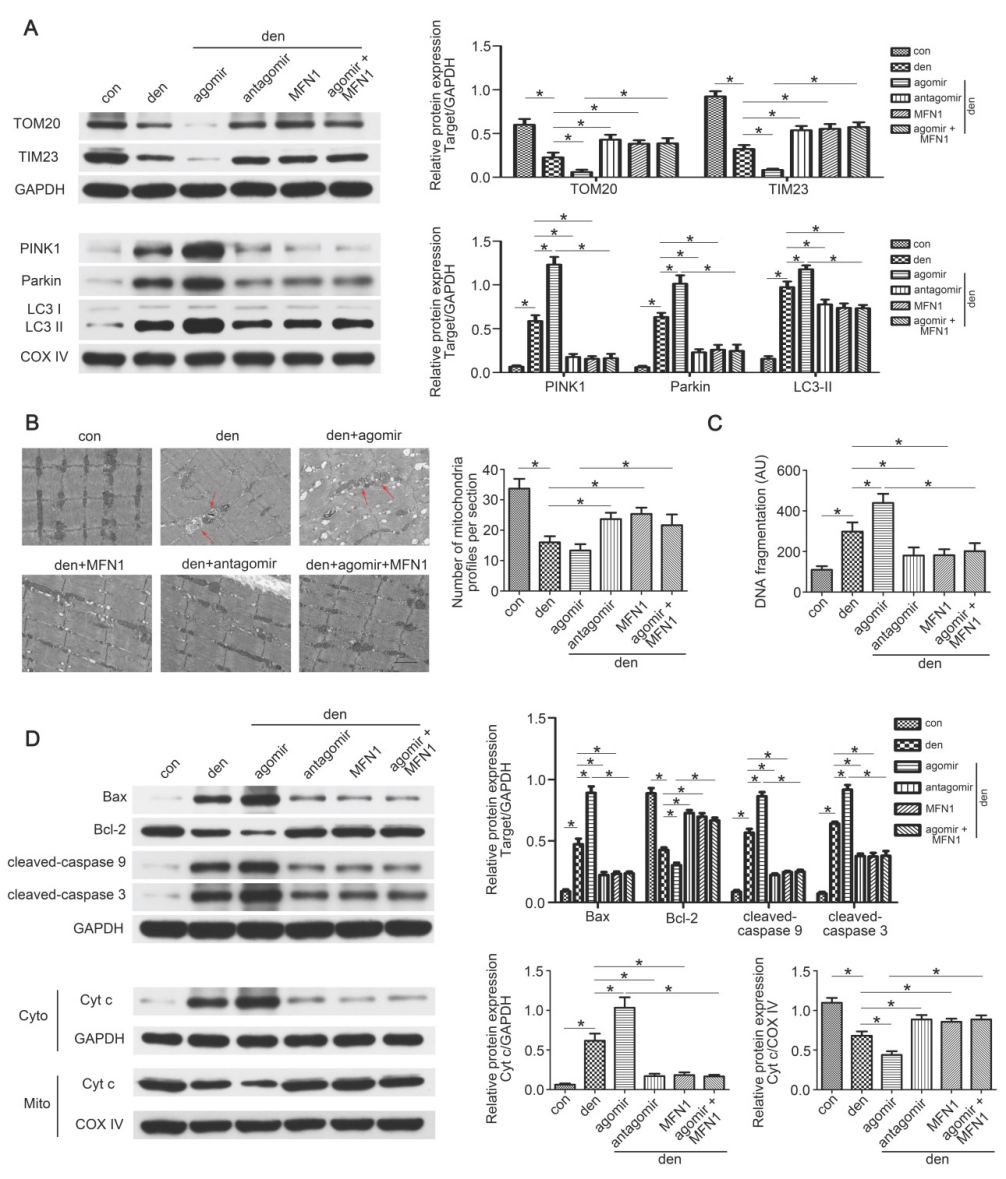
** Restoring MFN1 expression attenuated miR-142a-5p induced mitophagy and mitochondria-mediated apoptosis in denervated gastrocnemius.** (A) Western blotting analysis of the mitophagy parameters in gastrocnemius. The expressions of total TOM20, TIM23 and mito-PINK1, Parkin, LC3II were evaluated separately. GAPDH and COX IV were used as internal references. (B) Microstructure of gastrocnemius and mitochondria was observed by TEM. Red arrows indicated autophagosomes enclosing mitochondria. The number of mitochondrial profiles were statistically analysed. Scale bar 1.25 μm. (C) Apoptosis in gastrocnemius was assessed by detecting DNA fragmentation. (D) Western blot analysis of the protein changes related to mitochondria-mediated apoptosis. Cytochrome c in cytosolic and mitochondrial subfractions was assessed respectively. Data were presented as mean ± SD. n=5. *P < 0.05. Den, denervation; Con, control; Cyto, cytoplasm; Mito, mitochondria; Cyt c, cytochrome c.

As the in vitro experiments had demonstrated the regulatory role of miR-142a-5p/MFN1 axis in mitophagy and apoptosis, we further explored this effect in denervated gastrocnemius. Western blot revealed that total TOM20 and TIM23 levels decreased whereas mito-LC3II, PINK1 and Parkin increased after denervation, and it became more pronounced upon miR-142a-5p agomir administration, indicating the activating of mitophagy. This trend was partly reversed by miR-142a-5p antagomir and rAAV-MFN1 (Figure 9A). Subsequently, TEM wax performed to observe the detailed changes of mitochondria. Obvious decrease of mitochondria was found in denervated gastrocnemius with or without miR-142a-5p agomir administration, showing more mitophagosomes (marked by red arrows). miR-142a-5p antagomir and rAAV-MFN1 administration partly restored the number of mitochondria in denervated gastrocnemius. Furthermore, rAAV-MFN1 injection reversed the changes of mitochondria induced by miR-142a-5p agomir, although the mitochondria number was still less than control (Figure 9B). Cell death ELISA was then conducted to quantify apoptosis in each group. A significant increase of DNA fragmentation was detected in denervated gastrocnemius, and this was aggravated by miR-142a-5p agomir, whereas miR-142a-5p antagomir and rAAV-MFN1 administration reversed this denervation-induced apoptosis. rAAV-MFN1 administration was even able to rescue cell apoptosis induced by denervation + miR-142a-5p agomir (Figure 9C). Western blot analysis was further performed to explore mitochondria-mediated apoptosis signal pathway. Consistent with our in vitro results, miR-142a-5p agomir aggravated denervation-induced increase of proapoptotic proteins, decrease of anti-apoptotic protein and release of cytochrome c from mitochondria into cytoplasm, which was reserved by rAAV-MFN1 administration (Figure 9D). Totally, these results demonstrated the regulatory effect of miR-142a-5p/MFN1 axis on mitophagy and apoptosis in denervated gastrocnemius.

## Discussion

Peripheral nerve injuries are a growing topic of interest, particularly in developing countries where workers often suffer disabilities stemming from peripheral nerve injury complications such as limb weakness or muscle atrophy. The peripheral nerves undergo a series of complex pathological changes (Wallerian degeneration) after nerve trauma, during which the distal axons degenerate and Schwann cells proliferate to direct axonal regrowth and remyelination 36, 37. Though in situ suture, autologous and allogenic nerve transplantation have been applied to repair peripheral nerve transection, a long time is necessary for the axons to regrow and reinnervate their targets, during which the denervated skeletal muscles decrease contractile activities and undergo atrophy gradually, leading to an unsatisfactory prognosis 1,38,39. As such, maintaining muscle mass before reinnervation is vital for the treatment of peripheral nerve injuries and is also the main purpose of this research.

In consistence with previous researches 40, 41, we found that the denervated skeletal muscle atrophied over time in a biphasic manner, showing a rapid loss of muscle mass over the first 2 weeks and then a more gradual reduction over the following 2 weeks, so we focused on the early stage of skeletal muscle atrophy in order to seek out the specific mechanism underlying this process. Morphological observation of the atrophied gastrocnemius revealed clear decreases in mitochondrial numbers as well as mitophagy activation, which inspired us and led us to focus on mitochondrial dynamics.

Mitochondria are dynamic organelles that constantly undergo fusion and division processes to maintain their proper morphology essential for their normal functions 8. Mitochondrial fusion is the union of two mitochondria, which is mediated by membrane-anchored proteins, mitofusin (MFN)-1,2 and optic atrophy (OPA)-1. MFN1 and MFN2 are located on the outer mitochondrial membrane and are involved in early steps of membrane fusion 42, 43, whereas OPA1 is associated with the inner membrane and is essential for inner membrane fusion 44. In addition to morphology control, mitochondrial fusion is essential to mitochondrial function 45. Mice lacking MFN1/2 in skeletal muscle exhibit both mitochondrial dysfunction and profound muscle atrophy 46. Meanwhile, mitochondrial dynamics also regulate cell apoptosis. Overexpression of MFN1 and MFN2 facilitates mitochondrial fusion and maintain mitochondrial function, while knocking down of MFN1 and MFN2 expedites cell apoptosis 34, 35. Herein, we found that the expression of MFN1 in denervated gastrocnemius was down-regulated by miR-142a-5p, resulting in mitochondrial fragmentation. Mitochondrial function was further suppressed by the miR-142a-5p/MFN1 axis, as evidenced by Δψm depolarization and OXPHOS inhibition. Furthermore, mitophagy and mitochondria-mediated apoptosis were both activated by the miR-142a-5p/MFN1 axis in denervated gastrocnemius.

Apoptosis is a cellular process that is conserved from worms to humans. It has been extensively studied, but primarily in mitotic cells/tissues. Apoptosis plays a crucial role in a variety of biological events, including embryonic development, tissue turnover and immunological defense 47, 48. In the past several studies had demonstrated the involvement of apoptosis, which was characterized by increases in TUNEL-positive cells, DNA fragmentation and BCL-2 family expression, in the loss of postmitotic skeletal muscle after denervation 41, 49. Here in the denervated gastrocnemius we consistently observed the increase of apoptosis (showing more DNA fragmentation). Considering the dysfunction of mitochondria in denervated gastrocnemius, we speculated that mitochondria-associated apoptotic signaling might be activated during muscle atrophy, and we confirmed this through subsequent experiments. Western blot analysis revealed the expression variations of apoptotic regulators in mitochondria-mediated apoptosis. In particular, Bax (a pro-apoptotic protein) was found to be up-regulated in denervated gastrocnemius, which could translocate to the mitochondria and expose its N-terminus via a conformational change upon induction of apoptosis. This conformational change allowed the Bax/Bax-homo-oligomerization and therefore insertion of Bax into the outer mitochondrial membrane. This was followed rapidly by the formation of a channel, and subsequent release of the mitochondria-resided apoptogenic factors (e.g. cytochrome c, AIF and Smac/DIABLO) into the cytosol 50, 51. Conversely, the anti-apoptosis protein, Bcl-2, which was capable of forming Bcl-2/Bax-heterodimers and preventing the process of Bax/Bax-homo-oligomerization 52, was down-regulated. After the release of cytochrome c from mitochondria into cytoplasm, caspase-9 mediated apoptosis by assembling an apoptosome complex through the interaction of procaspase-9 with Apaf-1, dATP/ATP, and mitochondrial released cytochrome c, which subsequently recruited procaspase-3 to the complex and efficiently activated caspase-3, a common effector caspase, by the caspase-9-mediated proteolytic cleavage and finally resulted in cell apoptosis.

Recently, mitophagy has been reported to play a beneficial role in eliminating damaged and unhealthy mitochondria and maintaining quality and quantity of the organelle 53. However, massive and persistent mitophagy leads to excessive degradation of mitochondria, which may contribute to a bioenergetic deficit and cell death 54, 55. PINK1/Parkin (PARK2) pathway mediated mitophagy regulates the autophagic removal of mitochondria. After depolarization of Δψm or accumulation of damaged mitochondria, PINK1 localizes to the mitochondrial outer membrane, where it promotes the recruitment of the E3 ubiquitin ligase Parkin to the mitochondria. Parkin promotes mitochondrial membrane protein ubiquitination. Then, mitochondria are wrapped by autophagosomes via an interaction between the autophagy receptor p62/SQSTM1 (Sequestosome 1) and LC3. Autophagosomes eventually fuse with lysosomes to form autolysosomes, which target mitochondria for autophagy clearance 56, 57. In the present research, PINK1/Parkin mediated mitophagy was observed to be activated in denervated gastrocnemius, and we determined that this process was regulated by the miR-142a-5p/MFN1 axis, resulting in significant reductions in mitochondrial number. Given the role of mitophagy dysregulation in various diseases, we therefore speculate that mitophagy may aggravate muscle atrophy by disturbing gastrocnemius energy supplies, impairing the maintenance of a normal cellular metabolism. Such a hypothesis can be confirmed through additional experiments aimed at blocking the mitophagy process.

However, interpretation of the role of MFN1 in specific fiber types seems complicated since the gastrocnemius used in our experiments is a mixed type muscle. Previous researches reported that mice lacking both MFN1 and MFN2 exhibited impaired mitochondrial fusion and profound muscle atrophy in TA (fast-twitch fibers) and soleus (slow-twitch fibers). In contrast, muscles carrying just one MFN allele showed much milder histological defects. Besides, MFN1 knockout achieved quite different results (more patches of abnormal mitochondria and more mtDNA deletions) from MFN2 knockout models 46, 58. Denervation leads to various pathological changes in muscles, such as protein homeostasis disorder, apoptosis and so on 1, which further complicate the comprehension of MFN1 in gastrocnemius. In addition, it is uncertain whether the miR-142a-5p/MFN1 axis is responsible for atrophy in other denervated muscles (except for gastrocnemius) given that different fibers and different muscles are known to respond differently to denervation 59, 60, further studies concerning the role of miR-142a-5p/MFN1 axis in various fiber types will be the object of our future studies.

Considering that the atrophy of skeletal muscle following denervation is a complicated process, no suitable in vitro model has yet been designed capable of replicating such a denervated state, C2C12 cells were therefore used for all in vitro studies to explore the effect of miR-142a-5p/MFN1 axis on mitochondrial function, apoptosis, and mitophagy. Moreover, it is difficult to use specific blockers to directly inhibit mitochondria-mediated apoptosis, mitophagy or improve mitochondrial function considering their complicated interaction, so we can't determine the dominant driver of gastrocnemius atrophy. Furthermore, we were not able to achieve complete recovery of muscle atrophy via targeting the miR-142a-5p/MFN1 axis, indicating that other mechanisms are likely involved in the atrophy process. Indeed, previous studies have shown that the ubiquitin-proteasome system contributes to protein degradation in denervation-induced muscle atrophy.

## Conclusions

In summary, our results demonstrate that miR-142a-5p/MFN1 axis mediates the atrophy of denervated skeletal muscle, in which mitochondrial dysfunction, mitophagy and apoptosis may play vital roles downstream. The present findings contribute previously unknown insights into the mechanisms underlying the role of mitochondrial dynamics in muscle atrophy. Besides, the findings provide evidence that targeting miR-142a-5p/MFN1 axis may be a therapeutic alternative to relieve skeletal muscle atrophy induced by denervation.

## Supplementary Material

Supplementary methods, figures and table.

## Abbreviations

MFN1: mitofusin-1
TA: tibialis anterior
TEM: transmission electron microscopy
EMSA: Electrophoretic Mobility Shift Assay
OXPHOS: oxidative phosphorylation
miRNAs: microRNAs
DMEM: Dulbecco's modified Eagle's medium
FBS: fetal bovine serum
HE: Hematoxylin-eosin
WGA: wheat germ agglutinin
PINK1: PTEN induced putative kinase 1
BCA: bicinchoninic acid assay
Δψm: mitochondrial membrane potential
ROS: reactive oxygen species
FCM: flow cytometry
OPA1: optic atrophy-1
