# Supplementary material for: Denervation drives skeletal muscle atrophy and induces mitochondrial dysfunction, mitophagy and apoptosis via miR-142a-5p/MFN1 axis
Source: Theranostics. 2020 Jan 1;10(3):1415–32. doi: 10.7150/thno.40857 (PMC6956801; doi:10.7150/thno.40857)
Supplement: Supplementary file 1 — Supplementary methods, figures and table. [file thnov10p1415s1.pdf]

Supplementary methods

Immunofluorescence

Paraffin-embedded gastrocnemius and TA were cross-sectionally cut into 4-μm sections as above. After antigen retrieval, permeabilization, and goat serum blocking, primary antibody (rabbit anti-WGA, 1:50; Abcam) incubation was conducted overnight at 4°C. Samples were then stained for 1 hour with secondary Alexa Fluor 488-conjugated secondary antibodies (1:300, Invitrogen, USA), followed by a 5 minutes DAPI/PI (Sigma, USA) staining. Samples were then imaged using a fluorescence microscope.

Quantitative real-time PCR (qRT-PCR)

For qRT-PCR, the total RNA from cells were isolated with the TRIzol reagent (Invitrogen). cDNA was synthesized using the M-MLV First Strand Kit (Takara, Japan) according to the manufacturer's instruction. The real-time PCR was performed on an ABI 7500 System (Applied Biosystems, Foster City, CA) by using the SYBR Green PCR mixture (Takara). U6 was used as internal control. The relative expression levels were calculated using the comparative Ct method. Primer sequences were as follows: miR-142a-5p, 5'-GGCCCATAAAGTAGAAAGCACTAC-3' (forward) and 5'-CTCAACTG GTGTCGTGGAGTC-3' (reverse); U6, 5'- CTCGCTTCGGCAGCACAT-3' (forward) and 5'- AACGCTTCACGAATTTGCGT -3' (reverse).

Autophagy detection with Ad-GFP-LC3B

C2C12 cells were placed into 6 well plates on sterile coverslips and 40 μL of Ad-GFP-LC3B was added per well. After 24 hours incubation, cells were used for subsequent experiments. Green puncta were detected using a fluorescence microscope, assessing at least 30 cells in each group.

TUNEL staining and flow cytometry

Apoptosis was measured by TUNEL staining and flow cytometry (FCM). TUNEL staining was detected by TdT enzyme according to the manufacturer's instructions, and TUNEL-positive percentage was calculated per section. Flow cytometry was performed by labelling Annexin V and propidine iodide (PI) according to manufacturer's instructions (BD Biosciences, USA). Data were analysed by FlowJo software. Cells were considered apoptotic if they were annexin V-positive/PI-negative, while double-positive cells reflected necrosis or late apoptosis.

Analyses for Total Antioxidant Capacity in gastrocnemius

GSH, MDA, and SOD activities were performed using the commercial assay kits (Solarbio, China) to detect total antioxidant capacity of gastrocnemius. Detailed operations were performed according to the manufacturer's instructions. Protein levels of the lysates were measured using the BCA assay.

Fluorescence in situ hybridization (FISH)

Biotin-labeled miR-142a-5p probe (5'-AGTAGTGCTTTCTACTTTA

TG-3’) were designed and synthesized by RiboBio. The probe signals were detected with a FISH Kit (RiboBio) according to the manufacturer’s instructions. Briefly, Paraffin-embedded skeletal muscles were cross-sectionally cut into 4-μm sections. After prehybridization in PBS, the samples were hybridized at 37 °C for 30 min in hybridization solution. Then, nuclei were stained with DAPI. Samples were finally imaged using a fluorescence microscope.

Supplementary figures

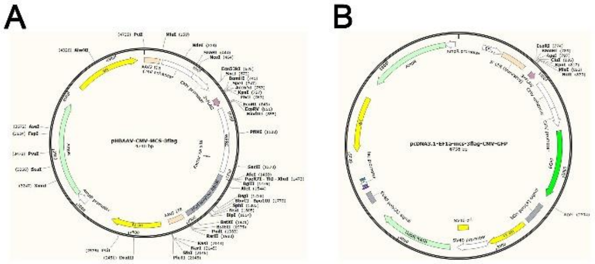

Figure S1. Structures of AAV vector and pcDNA 3.1 vector.

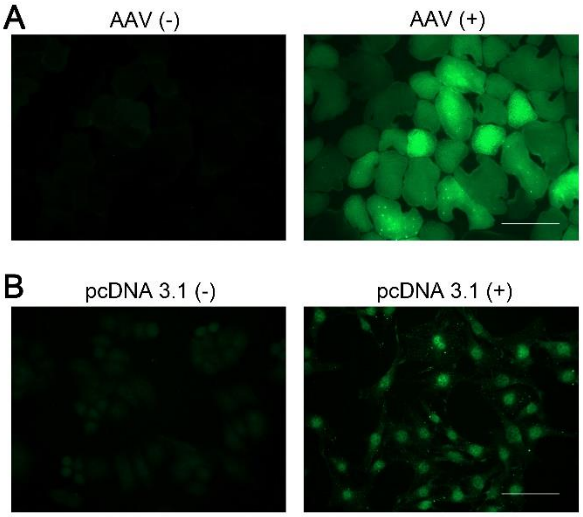

Figure S2. Validation of AAV and pcDNA 3.1 transfection. (A) Frozen sections of the gastrocnemius with or without AAV injection. Scale bar 50 μm.

(B) GFP fluorescence in C2C12 cells with or without pcDNA 3.1 transfection. Scale bar 50 μm.

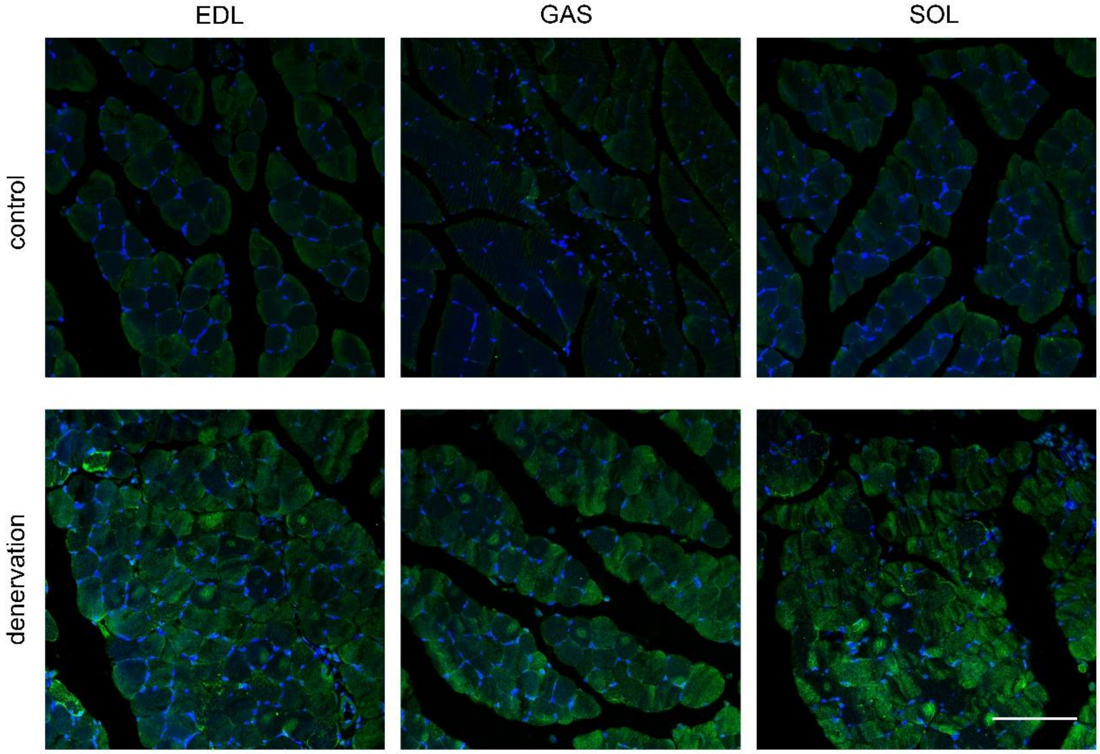

Figure S3. miR-142a-5p was up-regulated in extensor digitorum longus (EDL), gastrocnemius (GAS) and soleus (SOL) after denervation. Scale bar 50 μm.

Supplementary Tables

Table S1. List of differentially expressed miRNAs in denervated gastrocnemius

miRNA_ID	up/down	log2(foldchange)	P-value
mmu-miR-206-3p	up	2.6448	2.42E-11
mmu-miR-7213-5p	down	-6.737	7.16E-07

mmu-miR-128-2-5p	up	7.2062	1.85E-06
mmu-miR-181b-2-3p	down	-6.5236	2.98E-06
mmu-miR-193b-3p	down	-1.2844	3.86E-06
mmu-miR-129-5p	down	-1.2704	4.78E-05
mmu-miR-21a-5p	up	1.5837	0.00114665
mmu-miR-6540-5p	up	4.4351	0.001767353
mmu-miR-142a-5p	up	1.4452	0.003565333
mmu-miR-383-5p	down	-6.2606	0.003805175
mmu-miR-21a-3p	up	6.858	0.004122747
mmu-miR-205-5p	down	-2.9914	0.004997951
mmu-miR-138-2-3p	up	6.3219	0.005919043
mmu-miR-365-1-5p	down	-1.3389	0.006209496
mmu-miR-1949	up	6.0732	0.006767313
mmu-miR-5107-3p	up	6.2095	0.00753718
mmu-miR-1943-3p	down	-1.1807	0.007662521
mmu-miR-8114	up	1.6387	0.0084152
mmu-miR-34c-5p	up	1.4667	0.008887539
mmu-miR-3072-3p	down	-5.4812	0.009485467
mmu-miR-142a-3p	up	1.2742	0.010484091
mmu-miR-34b-3p	up	1.4892	0.019323154
mmu-miR-1963	down	-2.7188	0.020894042
mmu-miR-615-5p	down	-1.7438	0.021987399
mmu-miR-200b-3p	down	-1.6539	0.025576568
mmu-miR-203-3p	down	-1.0674	0.025613106
mmu-miR-218-5p	up	1.1942	0.030691946
mmu-miR-223-5p	up	1.1735	0.032935891

mmu-miR-455-5p	up	1.1314	0.033200399
mmu-miR-483-5p	down	-1.093	0.041387976
mmu-miR-21b	up	1.8621	0.042316892
mmu-miR-674-3p	up	1.0148	0.042595626
mmu-miR-34b-5p	up	1.4761	0.044150546
